# Thanks

**Published:** 1860-10

**Authors:** 


### Thanks.

The Library has been the recipient of some rare antique works during the past Summer. They are prized very highly, and for them the generous donors will please accept the grateful acknowledgments of those connected with the Institution.

The second edition of Wiseman's Surgery, presented by James McPherson & Co., Book-sellers, Atlanta, Ga., is a rare book. The work is entitled "Several Chirurgical Treatises; By Richard Wiseman, Serjeant Chirurgeon," and was printed in London, "Anno Dom., 1686." From the preface of the book it appears that the labors of the author were completed at least ten years before the publication of this edition, so that the book has been written not less than *one hundred and eighty-four years!* It was written under the reign of, and dedicated to "Charles II, King of Great Britain, France and Ireland, &c." The author's unique style may be seen in the following paragraph from the preface, or, as he terms it, "The Epistle to the Reader." He says:

"However, I resolving not to trust myself wholly with a case of this nice difficulty, did chuse a Friend to whose Judgment I did permit the whole. I mean the Learned Doctor Walter Needham of the Charter-house, who upon perusal of my papers was pleased to approve of my intention, and withal to alter what he thought fit in them, and always to cut off the Theory when it adventured upon Controversial Discourses; still urging, that this was not intended for a Book of Controversie, but of plain Institution; not to make men subtil Disputants, but good Practisers."

Another antiquated work, presented by Dr. Belton, is Scultetus' Surgery, in the Latin language, published two hundred and five years ago. It has forty-three plates showing the various Surgical Instruments then in use, and the manner of applying them in the treatment of Surgical diseases.

A mutilated copy of the Edinburgh Dispensatory, deposited in the Library by Mr. David Swobe, of Atlanta, has been so badly torn, the date of its publication cannot be ascertained, but from references in the body of the work to dates previous to the writing of the book, the inference is that it issued from the press over one hundred years ago.

In addition to the hundreds of volumes of modern scientific works derived from various sources, already in the Library, we are pleased to see, in a recent letter directed to the Dean of Atlanta Medical College, from Hon. L. J. Gartrell, Representative of the 4th Congressional District, that the Faculty are tendered 49 volumes, and that they will shortly be sent to the Library by his instruction. We learn that many of them are valuable scientific works, very appropriate for the College Library.

This donation, though valuable, is small compared with some of a similar character made to the Library previously. This gentleman's liberality toward the College, did not commence with his contributions to the Library. In the dark days of the Institution, when it was struggling to maintain its existence, under pecuniary embarrassment, his munificence was manifested. In behalf of the Institution, we tender unfeigned thanks for these repeated demonstrations of kindness and liberality.

We should be remiss and ungrateful did we fail, in this connection, to allude to other kind friends of Atlanta, who afforded substantial aid at a time when it was vitally important. Their names are held in grateful remembrance by those connected with the College.

After making these acknowledgments of individual contributions, we cannot close this article without referring again to the ample donation of the State in relieving the College entirely from pecuniary embarrassment.

